# Identifying Thrombus on Non-Contrast CT in Patients with Acute Ischemic Stroke

**DOI:** 10.3390/diagnostics11101919

**Published:** 2021-10-16

**Authors:** Shakeel Qazi, Emmad Qazi, Alexis T. Wilson, Connor McDougall, Fahad Al-Ajlan, James Evans, Henrik Gensicke, Michael D. Hill, Ting Lee, Mayank Goyal, Andrew M. Demchuk, Bijoy K. Menon, Nils D. Forkert

**Affiliations:** 1 Department of Clinical Neurosciences, University of Calgary, Calgary, AB T2N 1N4, Canada; eqazi01@gmail.com (E.Q.); alexis.wilson1@ucalgary.ca (A.T.W.); connor.ccm@gmail.com (C.M.); dr.f-alajlan@hotmail.com (F.A.-A.); james.w.evans1@gmail.com (J.E.); henrik.gensicke@usb.ch (H.G.); michael.hill@ucalgary.ca (M.D.H.); tlee@robarts.ca (T.L.); mgoyal2412@gmail.com (M.G.); ademchuk@ucalgary.ca (A.M.D.); docbijoymenon@gmail.com (B.K.M.); nils.forkert@ucalgary.ca (N.D.F.); 2 Hotchkiss Brain Institute, University of Calgary, Calgary, AB T2N 1N4, Canada; 3 Department of Radiology, University of Calgary, Calgary, AB T2N 1N4, Canada; 4 Department of Community Health Sciences, University of Calgary, Calgary, AB T2N 1N4, Canada; 5 Seaman Family MR Center, University of Calgary, Calgary, AB T2N 1N4, Canada; 6 Lawson Health Research Institute, Robarts Research Institute, Western University, London, ON N6C 2R5, Canada

**Keywords:** thrombus segmentation, hyperdense sign, Hounsfield units, non-contrast CT, thrombus, thrombus characteristics

## Abstract

The hyperdense sign is a marker of thrombus in non-contrast computed tomography (NCCT) datasets. The aim of this work was to determine optimal Hounsfield unit (HU) thresholds for thrombus segmentation in thin-slice non-contrast CT (NCCT) and use these thresholds to generate 3D thrombus models. Patients with thin-slice baseline NCCT (≤2.5 mm) and MCA-M1 occlusions were included. CTA was registered to NCCT, and three regions of interest (ROIs) were placed in the NCCT, including: the thrombus, contralateral brain tissue, and contralateral patent MCA-M1 artery. Optimal HU thresholds differentiating the thrombus from non-thrombus tissue voxels were calculated using receiver operating characteristic analysis. Linear regression analysis was used to predict the optimal HU threshold for discriminating the clot only based on the average contralateral vessel HU or contralateral parenchyma HU. Three-dimensional models from 70 participants using standard (45 HU) and patient-specific thresholds were generated and compared to CTA clot characteristics. The optimal HU threshold discriminating thrombus in NCCT from other structures varied with a median of 51 (IQR: 49–55). Experts chose 3D models derived using patient-specific HU models as corresponding better to the thrombus seen in CTA in 83.8% (31/37) of cases. Patient-specific HU thresholds for segmenting the thrombus in NCCT can be derived using normal parenchyma. Thrombus segmentation using patient-specific HU thresholds is superior to conventional 45 HU thresholds.

## 1. Introduction

The hyperdense sign is a marker of intra-vascular thrombus in non-contrast computed tomography (NCCT) in patients with acute ischemic stroke. A precise clot segmentation can be clinically important as it represents the basis for the assessment of thrombus morphometry, such as clot length. Riedel et al., for example, showed that a hyperdense artery sign in NCCT measuring >8 mm in length had a negligible chance of recanalizing with intravenous alteplase alone [1]. Likewise, Wu et al. reported that radiomics-based intracranial thrombus features, which require a detailed clot segmentation as the basis, were predictive of recanalization success with intravenous alteplase [2].

Manual clot segmentation is still the standard procedure for image-based clot analyses in research studies, but this approach is not feasible for clinical applications due to its high time requirements. Furthermore, it can be challenging to identify the exact margins of the hyperdense clot, for example, due to signal noise and partial volume effects, especially in the case of thick-slice NCCT datasets, leading to considerable inter-observer differences in manual segmentations [3,4]. Therefore, Riedel et al. [5] suggested an arbitrary selected threshold of 45 Hounsfield units (HU) for semi-automatic thrombus segmentation using region growing. However, there are multiple factors that can affect the density of clots, as measured in HU, including the presence of calcium in vessel walls and increased hematocrit [3,6,7]. To date, there is no well-justified single HU threshold described in the literature that has proven to enable a good thrombus differentiation from normal tissue parenchyma with high sensitivity and specificity.

The aim of this work was to determine optimal HU thresholds that differentiate the thrombus from surrounding brain tissue in NCCT. We also examined associations between HU thresholds that best differentiate thrombus and patient-level variables such as age, hematocrit, and CT parameters such as the slice thickness. Finally, we compared three-dimensional thrombus models generated using HU thresholds from the proposed model to those derived using a standard 45 HU threshold, using the thrombus seen in CTA as a gold standard.

## 2. Materials and Methods

Data used in this work originate from the ESCAPE trial, a multicenter prospective randomized controlled trial of endovascular stroke therapy compared to best medical care [8]. The ethics board at each site approved the trial. All patients enrolled in this study had large anterior circulation occlusions in baseline CTA (carotid T/L or M1 middle cerebral artery), were enrolled within 12 h of symptom onset, had baseline NIHSS > 5 and ASPECTS > 5, and at least moderate collaterals in CTA defined as the filling of >50% of the middle-cerebral-artery pial arterial circulation. For this post hoc analysis, only patients with MCA-M1 segment occlusions in CTA and thin-slice (≤2.5 mm) baseline NCCT were included. All NCCT datasets used in this work were acquired with an in-slice spatial resolution of 0.625 × 0.625 mm^2^ using 120 kEV tube potential. None of the patients included received contrast administration prior to CT image acquisition.

In order to derive patient-specific thresholds for clot segmentation, the CTA was registered to the NCCT using rigid transformation, linear interpolation, and optimization of the mutual information metric, implemented within a multi-resolution registration framework. After registration, the proximal and distal margins of the thrombus and its centerline were manually defined in the CTA and superimposed on the NCCT. This was necessary so that the exact margins of the thrombus could be visualized and superimposed on the NCCT to enable accurate HU value extraction of the thrombus.

**Figure 1:**
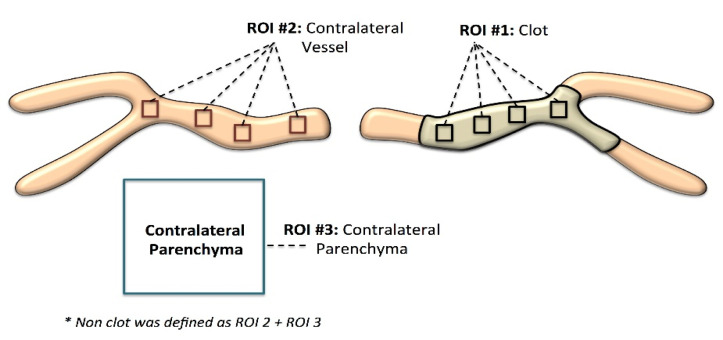
Regions of interest (ROIs) selected from three separate regions in the baseline NCCT: (1) thrombus: four 3 × 3 voxel ROIs were placed within the thrombus with the boundaries being determined using corresponding registered CTA datasets. (2) Contralateral artery: four 3 × 3 voxel ROIs were placed within the contralateral artery; the lumen of the artery was identified on the registered CTA and four 3 × 3 ROIs were placed along the center axis of the lumen. Hounsfield units were measured in the NCCT. (3) Contralateral parenchyma: a 10 × 10 voxel ROI was placed in the contralateral parenchyma in the NCCT.

Four regions of interest (ROIs; 3 × 3 voxels in size) were manually drawn at equidistant positions along the approximated centerline of the thrombus (Figure 1). The 3 × 3 voxel size was chosen because it allowed adequate sampling of the thrombus while avoiding including edges of the thrombus, which are often affected by partial volume effects. The registered CTA was also used to define four 3 × 3 ROIs in NCCT within the lumen of the contralateral vessel that corresponded to the thrombus location (normal vessel; Figure 1). Furthermore, a 10 × 10 voxel region was defined in normal appearing brain parenchyma adjacent to the contralateral artery segment on NCCT (normal brain tissue; Figure 1). To ensure standardization, this normal brain tissue ROI was placed within the white matter inferior to the contralateral MCA-M1. All ROIs were defined in the axial view.

Optimal HU thresholds that differentiated the thrombus from other non-thrombus tissues were calculated for each individual dataset using receiver operating characteristic (ROC) analysis by simultaneously maximizing sensitivity and specificity. Therefore, the NCCT HU attenuation values for 36 voxels (3 × 3 [ROI size] × 4 [number of ROIs]) in the thrombus, 36 contralateral vessel voxels (3 × 3 [ROI size] × 4 [number of ROIs]), and 100 (10 × 10 [ROI size]) contralateral parenchyma voxels were used for ROC analysis, whereas the NCCT HU values of the contralateral vessel voxels and parenchyma ROIs were combined to one class (non-thrombus). For ROC curve generation, the threshold used for segmentation was iteratively increased and the corresponding sensitivity and specificity separating thrombus and non-thrombus voxels were calculated. The optimal threshold was defined as the one that simultaneously maximized sensitivity and specificity.

Defining the precise thrombus ROI without any contributions from other tissues can be very challenging and time intensive, especially in the case of small clots. Thus, calculating the optimal HU threshold using the ROC analysis approach described above is not feasible for the analysis of larger databases or for a clinical routine. In contrast to this, the definition of a ROI in a normal vessel is easier but requires the registration of the CTA, which is also a time-consuming step and requires quality control of the registration results. Defining a ROI in normal brain tissue is even easier and faster and does not require registration of the corresponding CTA. Nevertheless, both approaches are generally feasible in a clinical context.

Therefore, linear regression was used to derive a general statistical model that best estimates the optimal patient–individual threshold as determined from the ROC analysis using “normal vessel” or “normal brain tissue” HU information and the other aforementioned variables (e.g., age and hematocrit). The theoretical benefit of such a model is that it only requires the definition of one region of interest (“normal vessel” or “normal brain tissue”) to estimate the patient-specific threshold.

**Figure 2:**
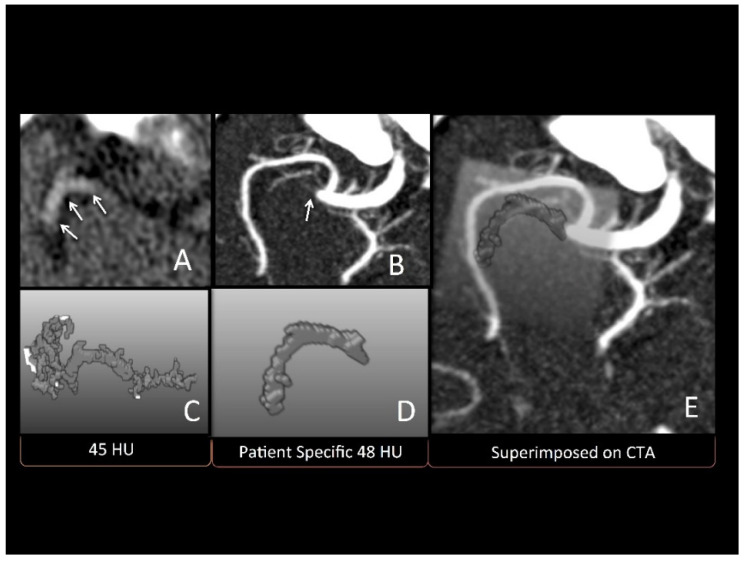
Patient with right distal middle cerebral artery M1 occlusion extending into the M2: (**A**) hyperdense sign in NCCT (marked by the arrow); (**B**) thrombus in baseline CTA; (**C**) three-dimensional model using conventional 45 HU threshold, which does not accurately depict CTA thrombus; (**D**) patient-specific threshold of 48 HU; (**E**) three-dimensional model using patient-specific HU threshold super-imposed onto CTA.

Finally, three-dimensional thrombus models were generated for the thrombus segmentations derived from very thin slice NCCT (≤0.625 mm) datasets using a volume-growing approach. Therefore, a rough bounding box was manually defined in the NCCT dataset, which encompassed the complete hyperdense artery sign, including a small safety margin. After this, an anisotropic smoothing filter was used to reduce noise artefacts while preserving important edge information of the thrombus structure. Multiple voxels were manually placed along the hyperdense artery sign and used as seed points for volume growing, employing either a fixed threshold of 45 HU or the estimated patient-specific threshold as the lower threshold (Figure 2). Thus, two different three-dimensional models were created for each patient, one with the standard 45 HU threshold, and the second using the patient-specific threshold estimated based on the statistical model derived above. The three-dimensional thrombus models were compared to the thrombus seen in CTA by two experts (JE and HG) in consensus. These experts were blinded to the method used to derive the models. An ordinal scale ranging from 0–4 was used to compare the three-dimensional thrombus models to the thrombus in CTA, as below:

- Three-dimensional model did not match the thrombus on CTA at all.
- Three-dimensional model approximated less than 50% of the thrombus on CTA.
- Three-dimensional model approximated 50–75% of thrombus on CTA.
- Three-dimensional model approximated 75–90% of thrombus on CTA.
- Three-dimensional model perfectly matched (90–100%) thrombus on CT.

In addition, the experts were also asked to give an overall impression which 3D model was a better match to the thrombus identified on CTA, or whether they were similar in quality.

Baseline characteristics are described using descriptive statistics. Optimal HU thresholds were derived at patient-level using logistic regression and ROC analysis, as described above. Pearson’s or Spearman’s correlation, as appropriate, was used to investigate the association between the optimal ROC-derived, patient-level HU thresholds and patient-level variables including age, hematocrit, slice thickness, HU in contralateral artery (using the mean of 4 ROIs), and average HU in normal brain parenchyma.

Linear regression was used to build statistical models that predicted the patient–individual optimal HU threshold for clot segmentation using variables that were identified as significant in the previous analysis. Assumptions of normality of residuals and heteroskedasticity were met for all statistical models that used linear regression. We compared these linear regression models to identify the best-performing model using Akaike (AIC) and Bayesian Information Criterion (BIC).

Finally, we compared the 3D thrombus models generated using the standard 45 HU and patient-level optimal HU threshold using non-parametric statistics.

All statistical analyses were performed using Stata (v. 13.0; Stata Corp LP, College Station, TX, USA).

## 3. Results

**Figure 3:**
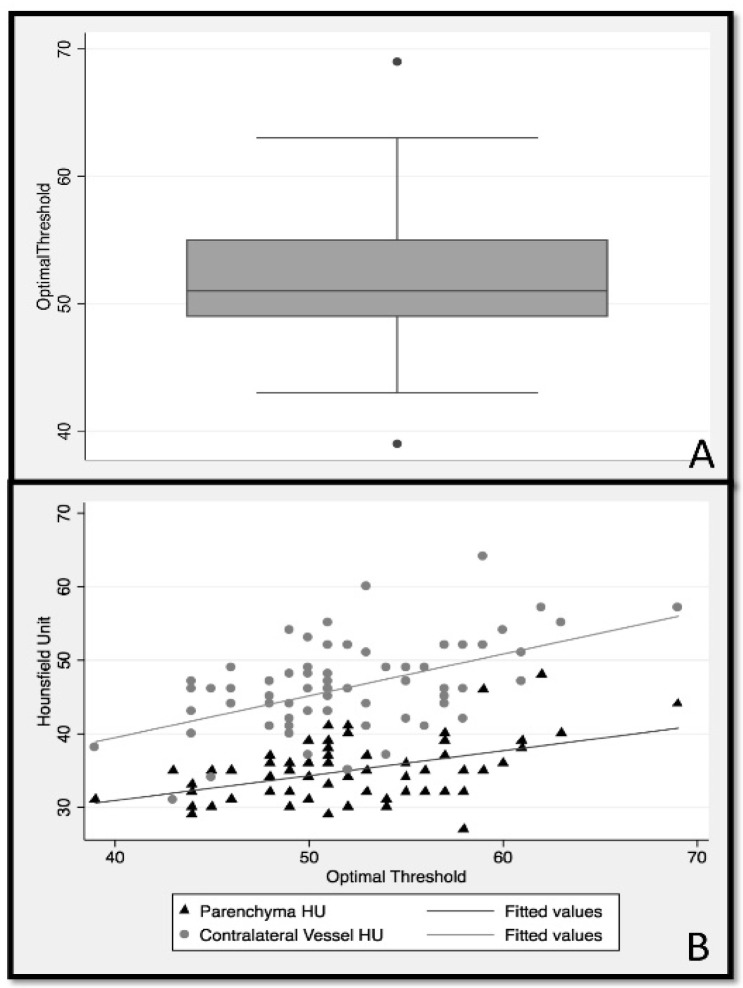
Panel (**A**) shows a box plot of the distribution of optimal thresholds that were calculated using ROC analysis comparing thrombus HU to normal tissue (parenchymal + contralateral vessel). A wide distribution indicates that there is no single HU threshold that is optimal to discriminate thrombus from normal tissue. Panel (**B**) is a two-way scatter plot showing that contralateral HU and parenchyma HU predict optimal HU threshold similarly.

Among 315 patients enrolled in the ESCAPE study, 70 patients with thin slice NCCT (≤2.5 mm) met the inclusion criteria (male sex 52.9%; median age 70; IQR 60 to 81 years). ROC analysis showed that the optimal HU threshold discriminating thrombus in NCCT from other non-thrombus tissues varied considerably between patients, with a median of 51 HU (IQR:49–55) (Figure 3A).

Testing for an association between clinical characteristics such as age and hematocrit, imaging characteristics including mean thrombus HU, mean contralateral artery HU, mean contralateral brain parenchyma HU, and slice thickness of NCCT revealed a modest positive correlation between patient hematocrit and contralateral artery HU (*r* = 0.43). Additionally, minor negative correlations were noted between slice thickness and ipsilateral thrombus HU (*r* = −0.25) and between slice thickness and contralateral artery HU (*r* = −0.22). No statistically significant correlation was noted between any other variables.

Linear regression (Table 1) used to determine optimal thresholds based on the average contralateral artery HU (model 1) accounted for 25% of the explained variability in optimal threshold (HU F (1.68) = 23.8, *p* < 0.001). The second linear regression model (Table 1) used the average contralateral parenchyma HU to predict the optimal threshold, which explained 21% of variability in optimal threshold (F (1.68) = 18.25, *p* < 0.001). Pearson correlation comparing the optimal threshold derived from model 1 with optimal threshold, as determined by the ROC analysis, was *r* = 0.46, while model 2 reached a correlation of *r* = 0.51. The AIC and BIC of models 1 (AIC = 421, BIC = 426) and 2 (AIC = 417, BIC = 422) were also similar. As such, the ability of average contralateral artery and contralateral parenchyma HU values to determine the optimal HU threshold for discriminating thrombus from other non-thrombus structures was similar (Figure 3B). Since it is clinically easier to define the contralateral parenchyma ROI compared to the contralateral artery ROI, the final analysis was conducted using only the contralateral parenchyma ROI for threshold evaluation.

**Figure 4:**
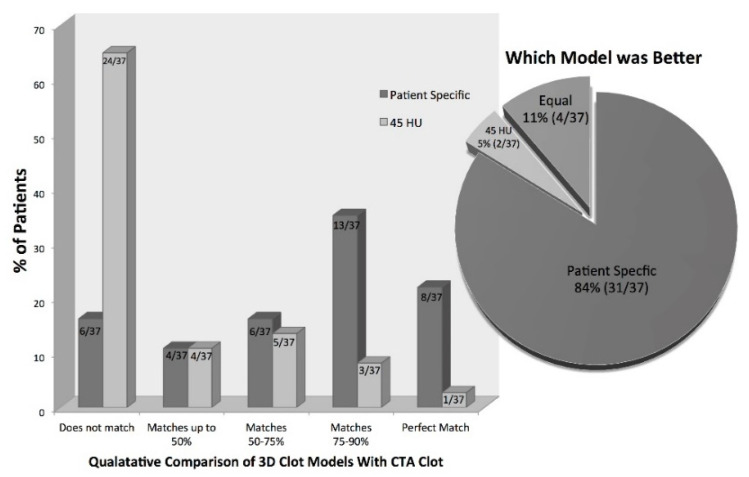
Qualitatively comparing three-dimensional models of the hyperdense thrombus in NCCT with CTA thrombus. Three-dimensional model formed using either (1) calculated patient-specific thresholds or (2) conventional single 45HU threshold.

For the qualitative analysis of the generated three-dimensional thrombus models, only patients with very thin slice NCCT (≤0.625 mm, *n* = 37) were used. In total, 73% (27/37) of the patients had three-dimensional thrombus models derived using our proposed statistical model that corresponded >50% to the thrombus on CTA. Compared to this, only 24% (9/37) of the three-dimensional thrombus models derived using the standard 45 HU threshold showed a correspondence >50% compared to the clot seen in CTA. Similarly, 22% (8/37) of patients had three-dimensional thrombus models derived using our proposed statistical model that matched perfectly with the thrombus in CTA compared to only 2.7% (1/37) of the models generated using the standard 45 HU threshold (Fischer’s exact test *p* < 0.01) (Figure 4). When asked which model was better, the experts chose the three-dimensional models derived using the proposed statistical model in 84% (31/37); however, the three-dimensional models generated using the standard 45 HU threshold were selected in 5% (2/37), and both models were judged as equal in 11% (4/37) (Figure 4).

## 4. Discussion

Identifying the hyperdense sign in NCCT is subjective and challenging. The results of this study show that there is considerable variation amongst patients in HU thresholds that best discriminate the thrombus from surrounding vessels and brain tissue (Figure 3). Using patient-specific thresholds, as proposed in this work, offers an easy approach that is suitable for clinical routine applications to differentiate the thrombus from surrounding tissue in CT head images from patients with acute ischemic stroke.

The statistical results revealed a modest correlation between patient hematocrit and contralateral vessel HU. It is likely that, as patient hematocrit rises (increased number of red blood cells per ml blood), HU within normal arteries increases. However, the HU of the thrombus itself may remain unchanged. This differential HU increase in arteries surrounding the thrombus may result in increasing complexity in differentiating thrombus from normal vessels in patients with higher hematocrit [7,9]. This also illustrates why there is not a single HU threshold that can discriminate the clot from the contralateral vessel and highlights the importance of determining patient-specific thresholds for this purpose.

Three-dimensional thrombus models can be created using patient-specific thresholds derived from information available within a patient’s CT scan (Figure 4). The results of this study suggest that 3D models generated using patient-specific thresholds more accurately discriminate the thrombus from other tissues when compared to a standard 45 HU threshold. This could be the reason why conventional methods of measuring thrombus length (whether defined as the hyperdense artery sign on NCCT or filling defect on CTA) have poor inter-rater and intra-rater reliability. Using calculated patient-specific thresholds to semi-automatically generate three-dimensional thrombus models requires only minimal user input. One only needs to place an ROI in the contralateral parenchyma and define seeds along the hyperdense vessel to generate a 3D model of the thrombus. The advantage of using such a semi-automatic method for thrombus segmentation over a completely manual thrombus segmentation is that it may generate more reproducible results needed for assessing important thrombus features in a clinical setting, including thrombus length and volume.

It should be mentioned that there are other complex methods for the segmentation of the thrombus in NCCT [10,11,12]. Some of these methods incorporate vessel characteristics and information about contralateral anatomy. It is possible that incorporating these parameters enables a more accurate thrombus segmentation. However, more complicated methods may also be less useable in the urgent clinical setting when time is an important factor. Our method for the segmentation of the thrombus using the contralateral parenchyma HU values to determine the optimal threshold that discriminates thrombus, and then using a volume growing approach to generate the 3D thrombus model, is simple and quick, and therefore more practical in the clinical setting. Our 3D models can potentially be used in conjunction with other tools in acute stroke imaging such as multiphase CTA color-maps and ColorViz for assessing collateral circulation [13,14].

This study has some limitations. Thrombus models were only generated for NCCT datasets with very thin slice thickness (0.625 mm) to minimize partial volume effect problems, especially affecting voxels in the periphery of the thrombus. While this allows one to build accurate high-resolution thrombus models, future studies should aim to test this method on thicker NCCT datasets as well as to generate three-dimensional models of thrombi from different vessels. In addition, we only included patients with MCA-M1 occlusions. Therefore, our findings cannot be applied to more distal occlusion sites. Inter-rater reliability of segmenting the thrombus models and comparing these models were not assessed in this study. We used consensus between two expert readers to assess the models, as our goal was to demonstrate proof of concept. This will be assessed in future studies.

## 5. Conclusions

Patient-specific thresholds best discriminate the hyperdense thrombus from surrounding tissue. These thresholds can be derived from contralateral parenchyma and used to generate accurate three-dimensional thrombus models. Ultimately, we envision that, with very little user input, clinicians will be able to not only reliably develop three-dimensional models of the thrombus but will also be able to automatically calculate thrombus properties instantly that can predict treatment success and guide decision making.

## Figures and Tables

**Table 1:** Linear regression models to predict optimal thresholds that discriminate clot from normal tissue using contralateral artery HU (model 1) and contralateral parenchyma (model 2).

	Model 1: Contralateral Artery HU	Model 2: Contralateral Parenchyma
Regression equation	Optimal threshold = 0.45 × contralateral artery HU + 31	Optimal threshold = 0.62 × parenchyma HU + 30.0
Significance	F (1.68) = 23.8, *p* = 0.0001	F (1.68) = 18.25, *p* = 0.0001
Degree of variability in optimal thresholds predicted by model	25%	21%

## Data Availability

The data presented in this study are available on request from the corresponding author. The data are not publicly available due to privacy reasons.
